# Radiomics in the evaluation of ovarian masses — a systematic review

**DOI:** 10.1186/s13244-023-01500-y

**Published:** 2023-10-02

**Authors:** Pratik Adusumilli, Nishant Ravikumar, Geoff Hall, Sarah Swift, Nicolas Orsi, Andrew Scarsbrook

**Affiliations:** 1 Department of Radiology, Leeds Teaching Hospitals NHS Trust, Leeds, UK; 2 Leeds Institute of Medical Research, University of Leeds, Leeds, UK; 3 West Yorkshire Radiology Academy, Level B Clarendon Wing, Leeds General Infirmary, Great George Street, Leeds, LS1 3EX UK; 4 Centre for Computational Imaging and Simulation Technologies in Biomedicine, University of Leeds, Leeds, UK; 5 Department of Medical Oncology, Leeds Teaching Hospitals NHS Trust, Leeds, UK; 6 Leeds Institute for Data Analytics, University of Leeds, Leeds, UK

**Keywords:** Ovarian neoplasms, Prognosis, Patient outcome assessment, Decision support techniques

## Abstract

**Objectives:**

The study aim was to conduct a systematic review of the literature reporting the application of radiomics to imaging techniques in patients with ovarian lesions.

**Methods:**

MEDLINE/PubMed, Web of Science, Scopus, EMBASE, Ovid and ClinicalTrials.gov were searched for relevant articles. Using PRISMA criteria, data were extracted from short-listed studies. Validity and bias were assessed independently by 2 researchers in consensus using the Quality in Prognosis Studies (QUIPS) tool. Radiomic Quality Score (RQS) was utilised to assess radiomic methodology.

**Results:**

After duplicate removal, 63 articles were identified, of which 33 were eligible. Fifteen assessed lesion classifications, 10 treatment outcomes, 5 outcome predictions, 2 metastatic disease predictions and 1 classification/outcome prediction. The sample size ranged from 28 to 501 patients. Twelve studies investigated CT, 11 MRI, 4 ultrasound and 1 FDG PET-CT. Twenty-three studies (70%) incorporated 3D segmentation. Various modelling methods were used, most commonly LASSO (least absolute shrinkage and selection operator) (10/33). Five studies (15%) compared radiomic models to radiologist interpretation, all demonstrating superior performance. Only 6 studies (18%) included external validation. Five studies (15%) had a low overall risk of bias, 9 (27%) moderate, and 19 (58%) high risk of bias. The highest RQS achieved was 61.1%, and the lowest was − 16.7%.

**Conclusion:**

Radiomics has the potential as a clinical diagnostic tool in patients with ovarian masses and may allow better lesion stratification, guiding more personalised patient care in the future. Standardisation of the feature extraction methodology, larger and more diverse patient cohorts and real-world evaluation is required before clinical translation.

**Clinical relevance statement:**

Radiomics shows promising results in improving lesion stratification, treatment selection and outcome prediction. Modelling with larger cohorts and real-world evaluation is required before clinical translation.

**Key points:**

• Radiomics is emerging as a tool for enhancing clinical decisions in patients with ovarian masses.

• Radiomics shows promising results in improving lesion stratification, treatment selection and outcome prediction.

• Modelling with larger cohorts and real-world evaluation is required before clinical translation.

**Graphical Abstract:**

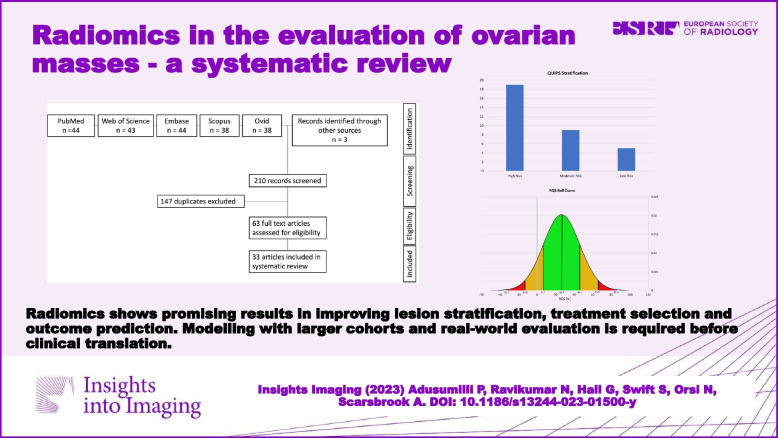

**Supplementary Information:**

The online version contains supplementary material available at 10.1186/s13244-023-01500-y.

## Introduction

Ovarian cancer (OC) is the leading cause of death from gynaecological cancer and the seventh commonest malignancy, accounting for 5% of all cancer deaths in women and 3% of overall cancer deaths [1–3]. Ovarian tumours include a heterogeneous group of benign, borderline and malignant lesions with variable morphology [4]. The high morbidity and mortality from OC stem from a lack of validated screening and the late presentation of non-specific symptoms [5–8].

Histopathological evaluation is the diagnostic gold standard. Tissue samples are in the main acquired during surgical staging whereas in other malignancies, biopsy diagnosis precedes further patient management. Imaging aims to differentiate benign adnexal lesions from malignancy.

First-line imaging of a clinically suspected ovarian mass usually involves trans-abdominal and trans-vaginal ultrasound assessment for suspicious features. The Risk of Malignancy Index (RMI) [9], Ovarian-Adnexal Reporting and Data System (O-RADS) [10], or International Ovarian Tumor Analysis (IOTA) [11] clinical support tools are often employed in conjunction with ultrasound assessment. RMI is a predictive model incorporating ultrasound features, menopausal status, and serum cancer antigen (CA-125) levels to estimate malignancy risk. O-RADS and IOTA, on the other hand, are categorisation systems that use ultrasound characteristics to stratify ovarian masses into different risk categories.

Staging is through contrast-enhanced computed tomography CT of the abdomen and pelvis. Magnetic resonance imaging (MRI) is used to characterise indeterminate ovarian lesions or confirm dermoid/endometriotic cysts identified on ultrasound scans. Fluorine-18 fluorodeoxyglucose positron emission tomography-computed tomography (FDG PET-CT) has a less established role in OC. Patients may undergo unnecessary or inappropriate surgery when non-invasive investigations are inconclusive; adverse consequences include disease progression, decreased fertility and premature menopause.

Radiomics involves the extraction of high-dimensional data from medical imaging, allowing quantitative analysis of the distribution and relationship of pixel levels [12]. This technique has been extensively studied in oncology for outcome prediction modelling [13–21]. The study aim is to appraise the published literature reporting application of radiomics to different imaging modalities in suspected OC, provide an overview of progress and remaining challenges in the field and outline future recommendations.

## Methods

### Search strategy and selection criteria

A search of MEDLINE/PubMed, Web of Science, Scopus, Embase, Ovid and ClinicalTrials.gov databases was performed in late March 2022. The criteria consisted of “radiomics” OR “radiomic” and “ovary” OR “ovarian”. Eligibility was assessed based on the title, abstract and subsequent full review. Articles not published in English, literature reviews and those assessing biopsy targets were excluded. References cited by articles were reviewed to identify further publications. Preferred Reporting Items for Systematic Reviews and Meta-Analysis (PRISMA) criteria were adhered to (Supplementary Files) [22].

Studies assessing biopsy targets were excluded from this review, focusing instead on articles describing the clinical utility of AI and radiomics in ovarian cancer diagnosis, prognosis, and treatment response prediction, which align with critical decisions made in multidisciplinary team (MDT) meetings. This emphasis on non-invasive assessment methods is particularly relevant, as biopsies are often avoided in clinical practice due to the risk of tumour seeding.

### Quality assessment

Validity and bias were assessed using the Quality in Prognosis Studies (QUIPS) tool [23] and Radiomic Quality Score (RQS) [12]. Two authors (P.A., A.S.) independently reviewed all studies with any discordance resolved in consensus.

QUIPS evaluates validity and bias and considers six areas (Supplemental Table S1). The overall risk of bias for each study was further categorised based on the following criteria: if all domains were classified as low risk, or there was up to one moderate risk, the research was classified as low risk of bias. If one or more domains were classified as high risk, the article was classified as high risk of bias. All studies in between were classified as having a moderate risk of bias [24]. Research with patient cohort sizes > 100 following exclusions was deemed to have had adequate participation.

RQS is a tool for assessing the quality and reporting of radiomic studies encompassing sixteen criteria with a maximum of thirty-six points (Supplemental Table S2). The RQS rewards or penalises a study’s methodology, analysis, and reporting.

## Results

### Literature search

**Fig. 1:**
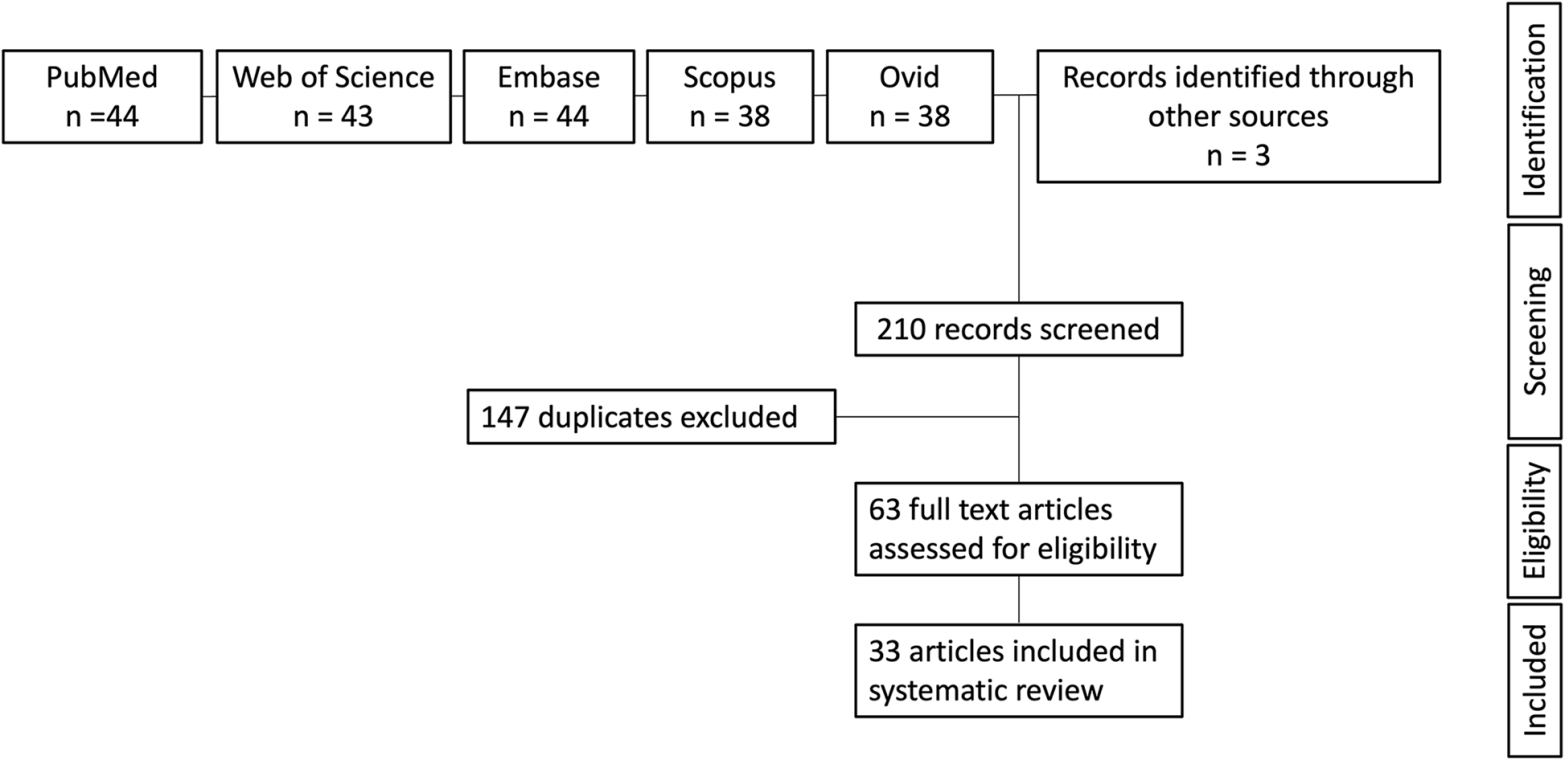
PRISMA flowchart

Database searches yielded a total of 207 articles. After eliminating 147 duplicates, 60 remained. In addition, 3 articles were identified from the references of these articles. All 63 were screened for eligibility, ultimately leaving 33 research studies (Fig. 1).

### Quality assessment

**Fig. 2:**
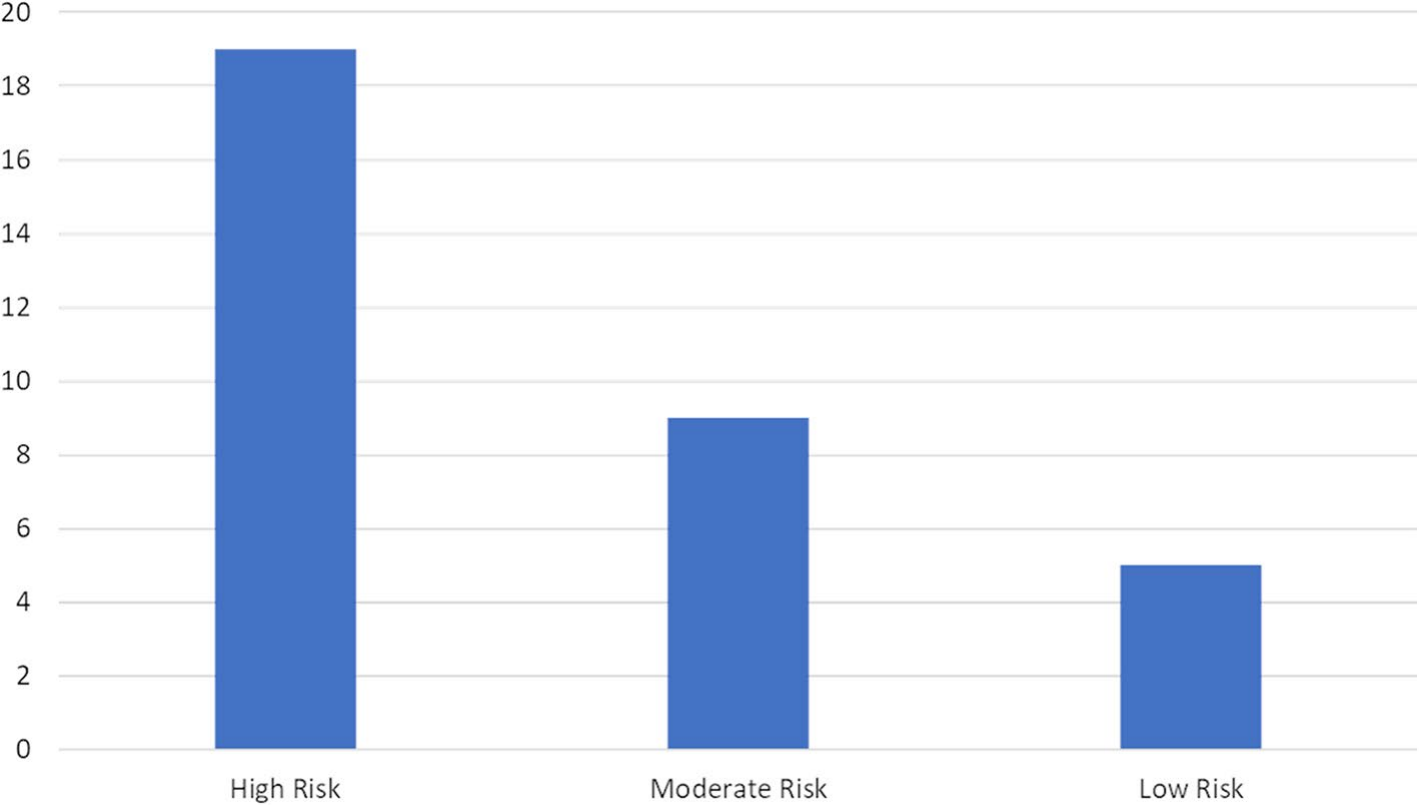
Quality in Prognosis Studies (QUIPS) stratification

One study demonstrated a low risk of bias in all six QUIPS domains [25], and 5/33 studies demonstrated a low overall risk of bias. Nineteen out of 33 studies had a high overall risk of bias, and 9/33 had a moderate overall risk of bias. Of the high-risk studies, six had a high risk of bias in participation, 16 in attrition, one in prognostic measurement, two in outcome measurement, 15 in confounding factors and five in analysis and reporting categories (Fig. 2 and Supplemental Table S3).

**Fig. 3:**
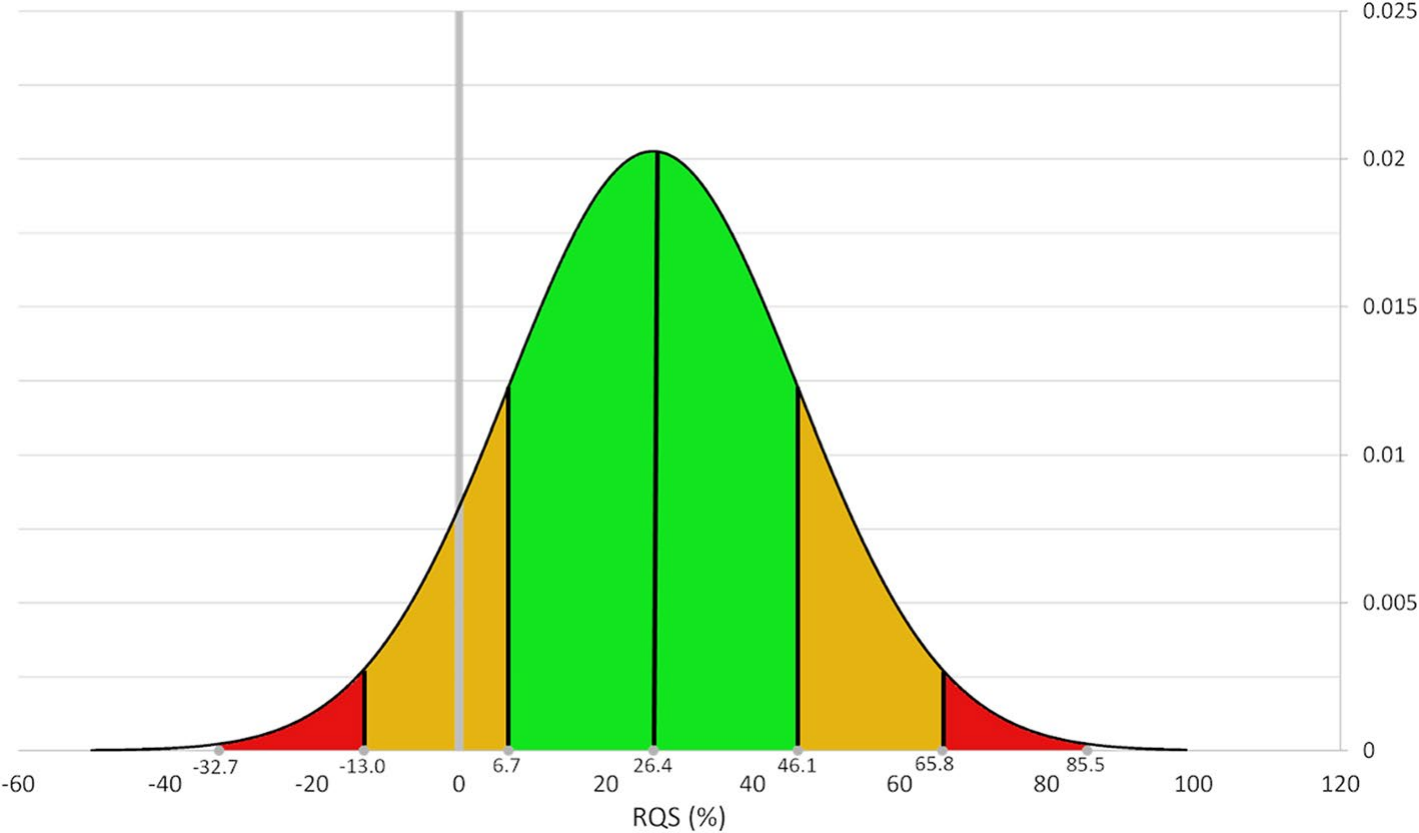
Radiomics Quality Score (RQS) bell curve

The highest RQS was 22/36 (61.1%), and the lowest score was − 6/36 (− 16.7%). Only one study had open-source code available. No studies had source imaging data or segmentations available in a repository. One study incorporated phantom study performance. No studies were prospective trials registered in a database (Fig. 3 and Supplemental Table S4).

### Outcomes

The analysed outcomes from these studies broadly fall into five main categories: diagnostic/pathological classification (15 articles), prognostication/outcome prediction (5 reports), treatment planning/monitoring (10 articles), metastasis prediction (2 studies), and a single study that assessed both diagnostic/pathological classification and prognosis prediction. Table 1 summarises the pertinent findings, while the following sections provide a detailed discussion of individual research studies, broken down by classification and imaging modality. Supplemental Table S5 provides an overview of different radiomic feature categories for reference and Supplemental Table S6 lists details of specific radiomic feature analysis performed in each of the studies.

**Table 1:**
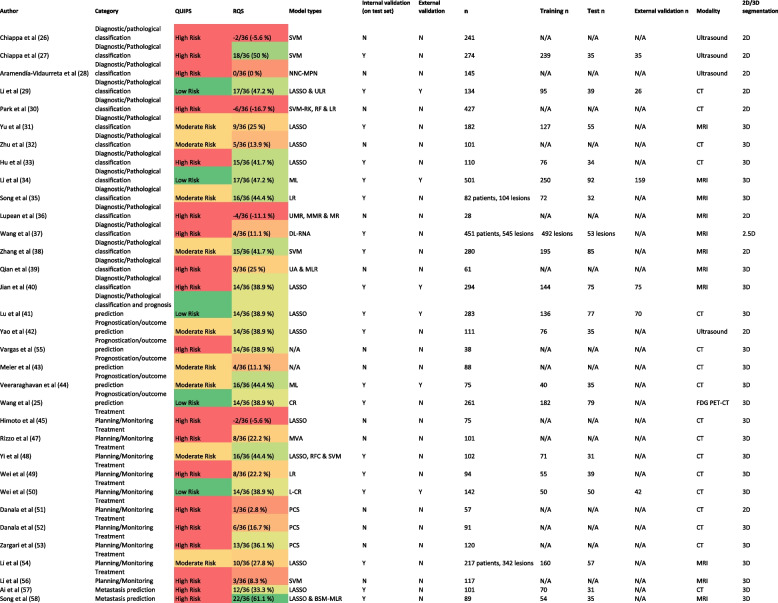
Summary of key features from the included studies [12, 23–54]

*BSM-MLR* backward stepwise multivariate logistic regression, *CR* Cox regression, *DL-RNA* deep learning-ResNet architecture, *L-CR* Lasso-Cox regression, *LASSO* least absolute shrinkage and selection operator, *LR* logistic regression, *ML* machine learning, *MLR* multivariate logistic regression, *MMR* multivariate multiple regression, *MR* multiple regression, *MVA* multivariate analysis, *NNC-MPN* Neural Network Classifier-Multilayer Perceptron Networks, *PCS* proprietary CAD scheme, *RF* random forest, *RFC* random forest classifier, *RK* radial kernel, *SVM* support vector machine, *UA* univariate analysis, *ULR* univariate logistic regression, *UMR* univariate multiple regression

In the QUIPS column: red, amber and green signify high, moderate and low risk respectively

In the RQS column, the lowest scores are red and this is a spectrum progressing through amber to green which signifies the highest scores

## Diagnostic/pathological classification

### Ultrasound

Chiappa et al. [26] developed a support vector machine (SVM) ultrasound model to predict malignancy risk in 241 masses. Model performance for solid malignant vs benign (accuracy 80.0%, AUC 0.87), cystic malignant vs benign (accuracy 87.0%, AUC 0.88) and motley (mixed solid and liquid) malignant vs benign (accuracy 81.0%, AUC 0.89) was good. A subsequent study [27] incorporated acoustic-shadow data and serum cancer antigen (CA-125) levels, yielding a better mean accuracy (91%) on prospective validation in a 35-patient test set.

Aramendía-Vidaurreta et al. [28] developed a multilayer perceptron ultrasound classification model utilising 79 radiomic features and age with 145 patients, demonstrating 98.8% accuracy, 98.5% sensitivity and 98.9% specificity (AUC 0.99).

### Computed tomography

Li and co-workers [29] developed a classification model based on contrast-enhanced CT in 134 lesions. The radiomic signature (RS) was calculated via a linear combination of selected radiomic features weighted by respective coefficients. Multivariate logistic regression (LR) combined clinical factors and RS to generate a nomogram demonstrating an externally validated AUC of 0.95.

Park and colleagues [30] developed a contrast-enhanced CT classification model in 427 patients with ovarian lesions. Radiomic features and patient age were used to create 3 ML models: SVM with radial kernel, random forest and LR. Respective sensitivities and specificities were 92.0% and 61.0% (AUC 0.87), 91.0% and 69.0% (AUC 0.88), and 92.0% and 61.0% (AUC 0.88).

Yu et al. [31] developed a SVM classification model using multiparametric CT to differentiate between serous borderline and serous malignant tumours in 182 patients. Arterial, venous, and equilibrium phase models achieved AUCs of 0.80, 0.86 and 0.73, respectively.

Zhu and colleagues [32] created a model based on non-contrast CT to differentiate ovarian epithelial carcinoma (OEC) and non-epithelial OC in 101 patients. The RS was a linear combination of selected radiomic features multiplied by respective calculated weights. The RS and clinical factors were combined with multivariable LR to derive a nomogram. The sensitivity and specificity for radiomics, clinical, and combined models were 94.0%, 47.0% (AUC 0.78); 72.0%, 87.0% (AUC 0.81); and 98.0%, 67.0% (AUC 0.87), respectively.

Hu et al. [33] developed a CT-based model to differentiate primary (POC) and secondary OC (SOC) in a study of 110 patients. The RS was a linear combination of selected radiomic features weighted by respective coefficients. The RS and clinical factors were combined in another model, and a nomogram was constructed. The combined model outperformed both radiomics and clinical models with 78.8% sensitivity and 90.7% specificity (AUC 0.75) in the validation cohort.

### Magnetic resonance imaging

Li and co-workers [34] subsequently developed a MRI-based machine learning (ML) model for the differentiation of borderline and malignant epithelial tumours was developed with single parametric and multiparametric LR models in 501 patients. The multiparametric solid-tumour model demonstrated the best externally validated performance (area under the receiver operating characteristic curve, AUC 0.90) and outperformed radiologists (AUC 0.80).

Song et al. [35] developed a MRI-based LR model to classify lesions as benign vs. borderline (task-A), benign vs. malignant (task-B), borderline vs. malignant (task-C), and benign vs. borderline vs. malignant (3-class classification) in 82 patients with 104 tumours. A multiparametric pharmacokinetic map was generated by combining important radiomic features from dynamic contrast-enhanced MRI. AUCs were 0.899, 0.865, and 0.893 for tasks A, B, and C, respectively. The 3-class classification task demonstrated AUCs of 0.893, 0.944, and 0.891 for the benign, borderline, and malignant groups, respectively. The pharmacokinetic model demonstrated an overall accuracy of 74.0% compared to radiologists at 64.4%.

Lupean et al. [36] developed regression models based on T2W-MRI to classify cysts as benign or malignant (84.6% sensitivity, 80% specificity, AUC 0.84) in 28 patients.

Wang and co-workers [37] developed a MRI deep learning (DL) classification model using 545 ovarian masses. radiomic features were harmonised and dimensionality reduced. The clinical model used LR, and seven radiologists independently interpreted the imaging. EfficientNet demonstrated the best accuracy (87.0%, AUC 0.81). While junior radiologists demonstrated 64.0% accuracy and senior radiologists achieved 74.0% accuracy, the former aided by the DL model achieved 77.0% accuracy.

Zhang and colleagues [38] developed a multiparametric MRI classification model to differentiate between malignant, benign, type I and II OECs and predict survival (training — 195 lesions, testing — 85 lesions). Survival analysis was undertaken with the least absolute shrinkage and selection operator (LASSO) regression to generate a risk score. The model demonstrated 90.3% accuracy (AUC 0.97) in classifying benign vs malignant and 92.7% accuracy (AUC 0.86) in classifying type I and II OECs. Radiologists demonstrated lower accuracy of 83.5%.

Qian and co-workers [39] developed MRI, clinical and combined models to classify type I and II OECs in 61 patients. Single sequence and multiparametric models were generated. The traditional model used univariate analysis of clinical factors, conventional MRI features and ADC values. Features with interclass correlation < 0.75 were included in multivariate LR. Traditional models demonstrated 91.0% accuracy (AUC 0.96). The performance of the mixed model was not significantly different (93.0% accuracy, AUC 0.91).

Jian and co-workers [40] developed a multiparametric MRI-based model to differentiate between type I and II OEC in 294 patients. The top four radiomic features were retained after feature elimination. A LASSO predictive model was generated for each sequence, a combined multiparametric model and a light combined model (FS-T2W, DWI, ADC). In the external validation cohort, the combined model demonstrated the best performance (AUC 0.85).

### FDG PET-CT

There were no studies identified supporting the role of FDG PET-CT in this domain.

### Summary

Several studies have investigated CT and MRI-based techniques, reporting diverse but generally promising results. Notably, a few studies reported ML models which outperformed radiologists, though the wider applicability of these findings requires additional validation. In ultrasound-based research, the integration of radiomics with clinical markers such as CA-125 levels has been explored for malignancy risk prediction. However, limitations across these studies, including lack of external validation, limited comparison with human expertise, and occasional lack of methodological detail, tempering the current findings. Additionally, small sample sizes and the absence of correction for multiple testing in certain studies present further challenges. These early findings underline the necessity for continued research, standardisation of methods, and rigorous validation to enhance the robustness and applicability of radiomics in ovarian cancer diagnosis and classification.

## Diagnostic/pathological classification and prognosis prediction

### Ultrasound

There were no studies identified supporting the role of ultrasound in this domain.

### Computed tomography

Lu et al. [41] developed a radiomics-determined mathematical descriptor of high-grade serous OC (HGSOC) risk phenotype using contrast-enhanced CT scans from 283 patients. A radiomics prognostic vector (RPV) was calculated using LASSO regression. High RPV was significantly associated with primary chemotherapy resistance, shorter progression-free survival (PFS), poor surgical outcome, ECM-receptor interaction, focal adhesions, fibronectin and a high proportion of tumour-associated stromal cells. DNA damage response pathways were found to be activated in RPV-low tumours.

### Magnetic resonance imaging

There were no studies identified supporting the role of MRI in this domain.

### FDG PET-CT

There were no studies identified supporting the role of FDG PET-CT in this domain.

### Summary

This model was developed using a sizeable patient cohort and validated with external data. The clear documentation of their method suggests the potential for reproducibility. Additional validation with larger datasets would strengthen its findings. The addition of MRI data to the model could provide a more comprehensive understanding of its applicability and performance.

## Prognostication/outcome prediction

### Ultrasound

Yao et al. [42] developed an ultrasound-based model to predict PFS in 111 patients. The RS was calculated through a linear combination of selected radiomic features weighted by respective coefficients. clinical factors and RS were incorporated to build a combined LASSO model which predicted 5-year PFS with 77.1% accuracy (AUC 0.83).

### Computed tomography

Vargas and co-workers [55] developed a contrast-enhanced CT-based model to evaluate associations between metastatic lesion inter-site tumour heterogeneity and clinical outcomes in 38 patients. Inter-site similarity matrices were calculated [43]. Inter-site similarity entropy, similarity level cluster shade, and inter-site similarity level cluster prominence were associated with reduced OS. Similarity level cluster shade, inter-site similarity level cluster prominence and inter-site cluster variance were associated with incomplete surgical resection. 19q12 involving cyclin E1 gene amplification (a primary oncogenic driver in HGSOC) occurred predominantly in patients with more heterogeneous inter-site textures.

Meier et al. [43] developed a model to assess inter-site texture homogeneity with survival and BRCA status using contrast-enhanced CT in 88 patients. Haralick features and pairwise similarities were calculated to generate the inter-site similarity matrix. Higher inter-site cluster prominence was associated with lower PFS, and higher inter-site entropy was correlated with lower OS. Higher values of all metrics were significantly associated with lower complete surgical resection in BRCA mutation-negative patients.

Veeraraghavan et al. [44] developed an integrated intra- and inter-site radiomics-clinical-genomic marker of HGSOC outcomes and explored the biological basis of radiomic features in 75 patients; based on their previous work [43, 55] and cluster dissimilarity (cluDiss) [45]. A clinical-genomic variables model demonstrated the best platinum resistance classification accuracy with an AUC of 0.78. CluDiss was found to be associated with PFS, negatively correlated with Wnt signalling (which controls cell proliferation) and positively correlated to several immune tumour micro-environment cell types [46].

### Magnetic resonance imaging

There were no studies identified supporting the role of MRI in this domain.

### FDG PET-CT

Wang and colleagues [25] developed a FDG PET-CT model for predicting PFS in HGSOC in 261 patients. Univariate Cox regression analysis was used to assess the correlation between radiomic features and PFS. PET and CT-RS were calculated from selected features weighted by regression coefficients. clinical factors were analysed with univariate Cox regression analysis. Multivariate Cox regression analysis was performed with different combinations of clinical, PET, CT and metabolic parameters. The combined clinical and PET-RS model showed the highest prognostic performance in the validation cohort (C-index 0.70, 95% CI 0.66–0.74).

### Summary

These studies provide valuable insights into the use of radiomics for predicting clinical outcomes in ovarian cancer, demonstrating the potential of integrated diagnostics combining imaging, clinical, and genomic data for guiding personalised decision-making. However, they are current limitations including relatively small sample sizes, absence of external validation, which could potentially result in overfitting and false positives. While the integration of multi-dimensional data in these models is promising, practical considerations regarding the availability of such data in various clinical settings also need to be addressed. Overall, the promising findings necessitate further robust research, with larger cohorts and rigorous statistical validation, before these models can be fully incorporated into clinical practice.

## Treatment planning/monitoring

### Ultrasound

There were no studies identified supporting the role of ultrasound in this domain.

### Computed tomography

Himoto et al. [45] developed a model to determine if tumour heterogeneity from baseline contrast-enhanced CT could identify patients who would benefit from immunotherapy in 75 women. Fewer disseminated disease sites and lower intra- and inter-tumoural heterogeneity were associated with durable clinical benefits. Shorter time to off-treatment was associated with more disease sites, presence of pleural disease/distant metastases, higher inter-tumoural heterogeneity, higher cluDiss and higher intra-tumoural heterogeneity. Higher inter-tumoural heterogeneity was also associated with a shorter time to off-treatment.

Rizzo and colleagues [47] developed a LR model to assess residual tumour and disease progression within 12 months after cytoreductive surgery using contrast-enhanced CT in 101 patients. Radiomic feature stability and reproducibility were assessed with phantom studies. The multivariate clinical-radiomics model (AUC 0.87) outperformed the clinical model (AUC 0.73).

Yi and co-workers [48] developed a model incorporating human sulfatase 1 single nucleotide polymorphisms (SNPs) and radiomic features from contrast-enhanced CT to predict platinum resistance in 102 patients. RS was calculated through a linear combination of selected radiomic features weighted by respective coefficients. The best performance in the validation cohort was achieved by the combined radiomic, clinicopathological, and SNP model (AUC 0.97).

Wei et al. [49] developed a LR model to predict 3-year post-operative recurrence of OC using contrast-enhanced CT in 94 patients. LASSO was utilised to build the RS which demonstrated 74.4% accuracy in the validation cohort (AUC 0.85). A subsequent model to predict recurrence risk was developed in 142 patients [50]. Kaplan–Meier analysis was performed, and patients were divided into high- and low-risk groups. A clinical prognostic model was constructed, and a nomogram based on combined radiomic and clinical factors was derived. The nomogram predicted 18-month and 3-year recurrence risks with accuracies of 84.1% and 88.9%.

Danala and co-workers [51] developed a model to identify features associated with predicting post-surgical chemotherapy response using contrast-enhanced CT in 57 patients. One model utilised pre-treatment CT and a second model utilised feature differences between pre- and post-treatment CTs. The top 15 radiomic features were combined with equal weights to generate a likelihood score. The fusion-based features achieved the best performance (AUC 0.84). This model was further refined in a subsequent study [52]. Twelve radiomic features were selected before applying a nearest-neighbour algorithm to select optimal features. The pre-treatment and feature-difference models demonstrated comparable AUCs of 0.81 and 0.83.

Zargari and colleagues [53] developed a model to investigate spatial and frequency domain features predictive of chemotherapy 6-month PFS using 120 patients. A generalised linear model achieved an AUC of 0.86.

### Magnetic resonance imaging

Li et al. [54] developed an MRI-based (T2W and CE-T1W) radiomic-clinical nomogram to predict residual disease (RD) status in 217 patients with HGSOC. LASSO was used to select radiomic features and build a single parameter signature to predict RD. Multivariable LR identified optimal radiomic and clinico-radiological features. The MF-based combined radiomic-clinical nomogram demonstrated the best performance with 73.6% accuracy (AUC 0.8).

Li et al. [56] developed a SVM model to predict recurrence risk in OC patients using MRI in 117 patients. L1 regularisation-based LASSO feature selection was used. A clinical model was developed using post-surgical residual tumour status and serum CA-125 levels. A fusion model incorporating CE-T1W, T2W and clinical factors demonstrated the best performance with 86.0% accuracy (AUC 0.86).

### FDG PET-CT

There were no studies identified supporting the role of FDG PET-CT in this domain.

### Summary

Overall, these studies showcase the application of radiomic models in assessing ovarian cancer treatment, with outcomes such as a residual tumour, disease progression, and platinum resistance. However, common limitations across these studies include a lack of robust internal or external validation and the need for larger datasets. Additionally, some studies, face potential compliance issues with non-Image Biomarker Standardisation Initiative (IBSI) compliant in-house software. Despite these challenges, the initial results indicate a promising direction for the field, emphasising the importance of continued research and validation efforts.

## Metastasis prediction

### Ultrasound

There were no studies identified supporting the role of ultrasound in this domain.

### CT

Ai and co-workers [57] developed a CT-based model to pre-operatively predict metastases in 101 patients. Optimal features were selected, and a RS was calculated using a linear combination of radiomic features weighted by respective weights. Univariate analysis was used to select clinical factors and build a LR model. A combined model with LR was built integrating radiomic and clinical factors (AUC 0.86).

### Magnetic resonance imaging

Song et al. [58] developed a model to predict peritoneal metastasis pre-operatively from multiparametric-MRI in 89 patients. RS was developed after feature elimination using LASSO and patients were stratified into low- or high-risk groups. A nomogram was constructed using multivariate LR with the RS and clinical factors. Radiologists demonstrated 65.7% accuracy (AUC 0.67) in the validation cohort. The RS demonstrated 80% accuracy (AUC 0.97) in the validation cohort, while the nomogram achieved 82.9% (AUC 0.94).

### FDG PET-CT

There were no studies identified supporting the role of FDG PET-CT in this domain.

### Summary

These studies highlight the promising potential of radiomics in predicting metastasis in ovarian cancer patients. The incorporation of both radiomic signatures and clinical factors in developing predictive models underscores their innovative approach. However, the relatively modest sample sizes utilised in both studies highlight the need for more expansive studies with larger datasets for robust validation. Notably, the absence of clear information on the use of CT contrast in the study by Ai et al. is a point that future research could clarify. Despite these challenges, these studies contribute valuable insights and provide a foundation for further research.

## Current limitations and future challenges

This systematic review highlights several areas of development for future radiomics research (Table 2).

**Table 2:** Limitations of the current literature and opportunities for the future

**Limitation**	**Opportunity**
1. Multiple scanners and imaging protocols	Phantom studies Image harmonisation techniques IBSI-compliant radiomic packages Validate reproducibility using publicly available data sets
2. Comparison with human readers	Undertake blinded review with human interpreters with varying experience levels
3. 2D segmentation and manual segmentation	Undertake all segmentation using 3D Develop automated/semi-automated segmentation techniques
4. Lack of external validation	Use publicly available data sets as external validation
5. Open source code	Upload code to public repositories such as GitHub

A significant limitation stems from inconsistent imaging protocols, feature extraction and feature selection methodologies and disparities between feature extraction packages. Radiomics involves quantitative analysis of image data, which is sensitive to variations in image quality, resolution, and contrast. Factors such as image acquisition parameters, reconstruction techniques, scanner manufacturers, and scan protocols can therefore introduce variability in the extracted features [59–63]. For CT, variations in kilovolt peak (kVp), tube current exposure time product (mAs), tube current modulation (TCM) settings, slice thickness, and reconstruction algorithms can introduce inconsistencies in radiomic analysis. Similarly, in MRI, magnetic field strength and pulse sequences affect the signal-to-noise ratio and contrast-to-noise ratio, which in turn influence radiomic features. Ultrasound imaging parameters, such as frequency, gain settings, and dynamic range, play a role in determining the appearance of tissue texture and the extraction of radiomic features. In PET-CT, the choice of reconstruction algorithm, standardised uptake value (SUV) measurements, time-of-flight (TOF) reconstruction, and point spread function (PSF) correction can all impact the consistency and comparability of radiomic features across different datasets. Ideally, studies should adopt standardised imaging protocols and feature extraction methodologies to minimise the impact of these variables. Phantom studies can help compensate for these variations to some extent; however, only one study utilised phantom studies to address scanner and protocol variability [47].

**Fig. 4:**
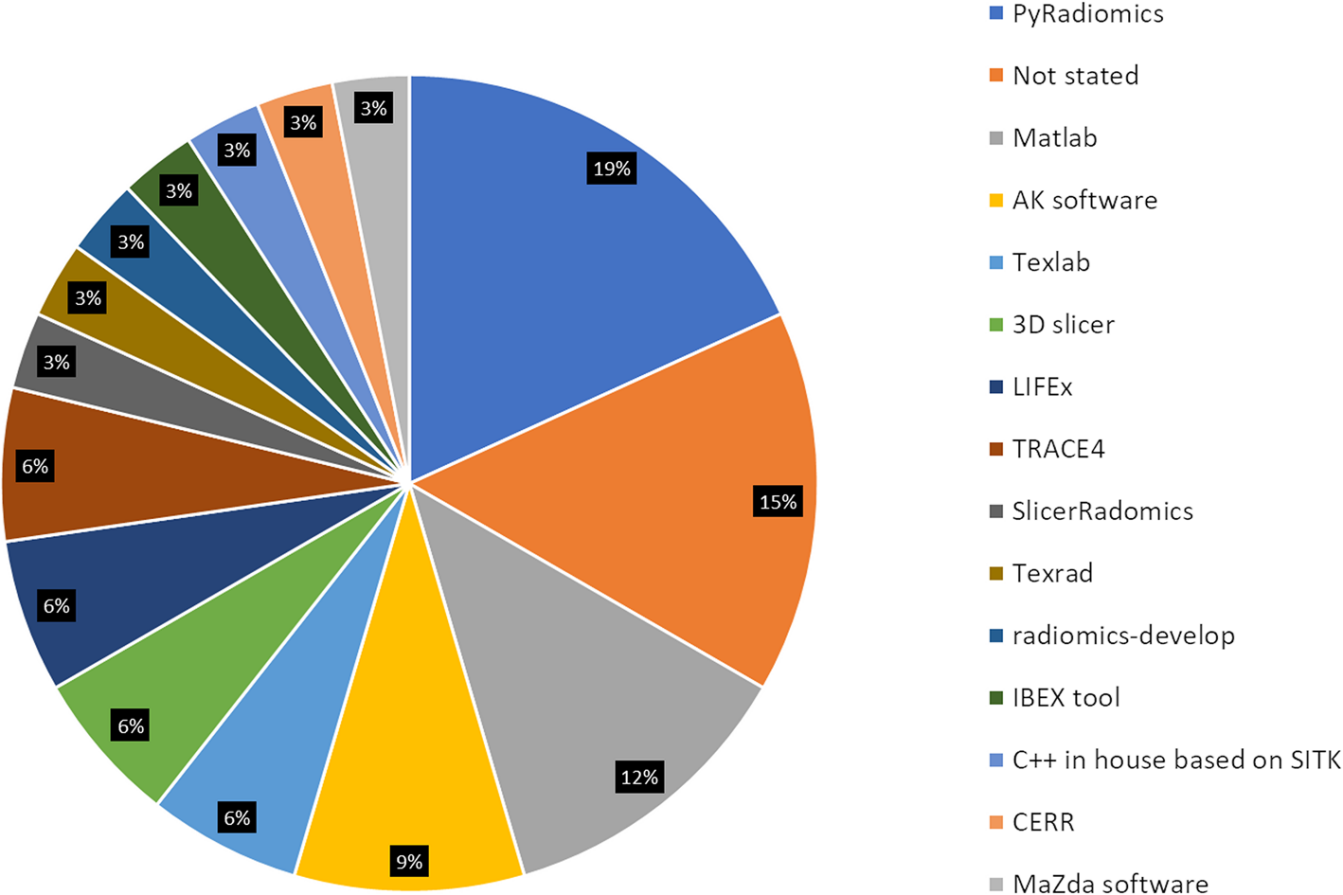
Radiomic packages used by the studies

It was not possible to identify any consistent trends in radiomic feature correlations, primarily due to the considerable variability in extraction methodologies, which encompassed a diverse array of radiomic packages, software tools, and parameter settings, ultimately leading to non-uniform feature extraction and limiting comparability of results across different studies. With a multitude of available radiomic software packages (Fig. 4), it is essential to ensure IBSI compliance [14, 64] to standardise the extraction process. Furthermore, some studies did not implement multiple testing correction when assessing the correlation between outcomes with radiomic features [30, 32, 36, 49] which increases the likelihood of false positives given the volume of features being analysed simultaneously.

Only 5/33 studies compared performance with radiologist interpretation [34, 35, 37, 38, 58]. This is crucial to enable a better understanding of the real-world potential and limitations of models. In this respect, Wang et al. [37] assessed the accuracy of radiologists assisted by AI, which is more representative of how these models would be employed clinically.

Variability in segmentation methodology was evident in the studies, as researchers employed different manual or automated segmentation techniques and opted for either 2D or 3D segmentations. A common drawback in these studies is the prevalent use of manual segmentation. All but one study employed a manual segmentation technique. One group developed a semi-automated technique utilising in-house software, although the code is not available for public review [51–53]. Manual and automatic segmentation methods present various advantages and disadvantages in the studies. Manual techniques allow for greater control and precision but are more labour-intensive and subject to inter- and intra-rater variability. Automatic or semi-automatic approaches offer increased efficiency and reproducibility, reducing both time taken and rater variability, but may potentially compromise accuracy. The labour-intensive nature of manual segmentation has resulted in relatively small cohort sizes in most studies, with sample sizes varying from 28 to 501 cases. This leads to further limitations, such as small internal and external validation cohorts. Out of the 33 studies, 14 did not include internal validation, and 27 lacked external validation.

The dimensionality of segmentation techniques exhibited considerable variation, with 9/33 (27.3%) studies using 2D segmentations rather than 3D volumes of interest (possibly due to time constraints). 3D segmentation provides a more comprehensive and accurate representation of tumour volume by capturing the tumour’s complete spatial context. This leads to reduced inconsistencies between slices, diminished inter/intra-rater variability, increased feature stability, and ultimately, more reliable and reproducible radiomic features. However, while 3D segmentation delivers superior tumour representation, it also presents challenges such as greater computational demands and extended processing times. These factors can impact the efficiency and scalability of research projects.

Ovarian lesions may demonstrate solid and cystic components. Included studies demonstrated variability in segmentation methodology, with only 2/33 (6.1%) studies investigating separate models based on solid, cystic and whole lesion segmentation. Deciding between the segmentation of the solid, cystic part or whole tumour, and whether to consider only the ovarian lesion or all lesions including disseminated disease, greatly influences extracted radiomic features and will affect the generalisability of models based on these features.

AI techniques have the potential to alleviate the challenges associated with manual segmentation and 3D processing. By automating the segmentation process, AI and DL can significantly reduce the time and labour required, ultimately leading to larger cohort sizes and more robust validation. Furthermore, advanced DL techniques could potentially bypass the need for segmentation altogether by directly learning and extracting radiomic features from raw imaging data. As AI models continue to advance, they may also demonstrate increased computational efficiency thereby overcoming computational and processing time limitations.

Code should be made publicly available, allowing other researchers to fine-tune models, for auditing and research transparency. Yet, only one study had easily accessible code available [37, 65]. Finally, larger studies are needed to ensure generalisable patient models.

## Conclusion

Radiomics has potential as a clinical diagnostic tool in patients with ovarian masses where it may allow better lesion stratification and inform future personalised patient care. Standardisation of feature extraction methodology, larger and more diverse patient cohorts and real-world evaluation are all required before clinical translation.

### Supplementary Information

**Additional file 1.** PRISMA 2020 Checklist.

**Additional file 2: Supplementary Table S1.** Quality in Prognosis Studies (QUIPS) criteria.

**Additional file 3: Supplementary Table S2.** Radiomics Quality Score (RQS) criteria

**Additional file 4: Supplementary Table S3.** Quality in Prognosis Studies (QUIPS) scoring for the assessed studies

**Additional file 5: Supplementary Table S4.** Radiomics Quality Score (RQS) scoring for the assessed studies.

**Additional file 6: Supplementary Table S5.** Radiomic feature categories. GLCM, Gray-level co-occurrence matrix; GLRLM, Gray-level run length matrix; GLSZM, Gray-level size zone matrix; GLDM, Gray-level dependence matrix; NGTDM, Neighbourhood gray-tone difference matrix; GLDM, Gray-level dependence matrix; LBP, Local binary patterns; ROI, Region of interest.

**Additional file 7: Supplementary Table S6.** Radiomic model details for studies. 3D, Three-Dimensional; ADC, Apparent Diffusion Coefficient; CA-125, Cancer Antigen 125; CA-153, Cancer Antigen 153; CA-199, Cancer Antigen 199; CE-T1WI, Contrast-Enhanced T1-Weighted Imaging; CEA, Carcinoembryonic Antigen; CER, Contrast enhancement ratio; Coif, Coiflets Wavelet Kernel; CP, Cluster Prominence; CT (radiomic feature), Cluster Tendency; CT (modality), Computed Tomography; DCE-MRI, Dynamic Contrast-Enhanced Magnetic Resonance Imaging; DCT, Discrete Cosine Transform; DE, Dependence Entropy; DV, Dependence Variance; DWI, Diffusion-Weighted Imaging; DWI FS, Diffusion-Weighted Imaging with Fat Suppression; F1, Feature 1; F2, Feature 2; F4, Feature 4; FIGO, International Federation of Gynecology and Obstetrics; FIRSTORDER, First Order (refers to a set of basic statistics features in radiomics); FOS, First Order Statistics; fPV, Fractional plasma volume; FS, Fat Suppression; FS-T2WI, Fat Suppressed T2-Weighted Imaging; GLCM, Gray Level Co-occurrence Matrix; GLCM-E, GLCM Energy; GLDM, Gray Level Dependence Matrix; GLN, Grey Level Nonuniformity; GLRLM, Gray Level Run Length Matrix; GLSZM, Gray Level Size Zone Matrix; GLVariance, Gray Level Variance; GLZLM, Gray Level Zone Length Matrix; HC, Haralick Correlation; HDLC, High Density Lipoprotein; HH, High-High; HHH, High-High-High; HHL, High-High-Low; HILAE, High intensity Large Area Emphasis; HISTO, Histogram; HL, High-Low; HLL, High-Low-Low; IAUGC, Initial area under the gadolinium concentration curve; IDM, Inverse Difference Moment; IDMN, Inverse Difference Moment Normalized; IDN, Inverse Difference Normalized; Imc1, Inverse Difference Moment Normalized; IMC2, Information Measure of Correlation 2; IQR, Interquartile Range; ISM, Inter-site Similarity Matrix; IV, Intensity Variability; K, Kurtosis; Kep, Rate constant; Ktrans, Volume transfer constant; LAHGLE, Large Area High Gray Level Emphasis; LALGLE, Large Area Low Gray Level Emphasis; LDHGLE, Large Dependence High Gray Level Emphasis (feature); LDLC, Low Density Lipoprotein; LGLE, Low Gray Level Zone Emphasis; LGLRE, Low Grey Level Run Emphasis; LH, Low-High; LHH, Low-High-High; LHL, Low-High-Low; LL, Low-Low; LLH, Low-Low-High; LLL, Low-Low-Low; LoG, Laplacian of Gaussian; LRE, Long Run Emphasis; LRLGLE, Long Run Low Grey Level Emphasis; MCC, Maximal Correlation Coefficient; MD, Mean Deviation; MI, Max intensity; MPP, Mean of Positive Pixels; MR, Magnetic Resonance; NGLDM, Neighborhood Gray Level Dependence Matrix; NGTDM, Neighboring Gray Tone Difference Matrix; PD12, Progression Disease 12; PET, Positron Emmision Tomography; PM, Peritoneal Metastasis; PostC, Post-contrast; RLNonUniformity, Run Length Non-Uniformity; RMS, Root Mean Squared; RP, Run Percentage; RT, Radiation Therapy; SALGLE, Small Area Low Gray Level Emphasis; SCP, Inter-site Cluster Prominence; SCV, Inter-site Cluster Variance; SD, Standard Deviation; SE, Inter-site Entropy; SHAPE, Shape-based feature; SRE, Short Run Emphasis; SRHGE, Short-Run High Gray-Level Emphasis; SRHGLE, Short Run High Gray Level Emphasis; SSF, Spatial Scale of the Filter; ST, Solid Tumor; SZLGE, Small Zone Low Gray Level Emphasis; SZNonUniformity, Size Zone Non-Uniformity; T1WI, T1-Weighted Imaging; T2WI, T2-Weighted Imaging; T2WI FS, T2-Weighted Imaging with Fat Suppression; TCHO, Total Cholesterol; TG, Triglyceride; Ve, Extravascular extracellular volume fraction; WAV, Wavelet Transform; WT, Whole Tumour; ZSV, Zone Size Variance; ZV, Zone Variance.

## Data Availability

The datasets used and/or analysed during the current study are available from the corresponding author upon reasonable request.

## Abbreviations

ADC: Apparent diffusion coefficient
AUC: Areas under the receiver operating characteristic curve
CA-125: Cancer antigen 125
CE-T1W: Contrast-enhanced T1 weighted
cluDiss: Cluster dissimilarity
CT: Computed tomography
DL: Deep learning
DWI: Diffusion-weighted imaging
FDG PET-CT: Fluorine-18 fluorodeoxyglucose positron emission tomography-computed tomography
FS-T2W: Fat saturated T2 weighted
HGSOC: High-grade serous ovarian cancer
IBSI: Image Biomarker Standardisation Initiative
IOTA: International Ovarian Tumor Analysis
LASSO: Least absolute shrinkage and selection operator
LR: Logistic regression
MDT: Multidisciplinary team
ML: Machine learning
MRI: Magnetic resonance imaging
O-RADS: Ovarian-Adnexal Reporting and Data System
OC: Ovarian cancer
OEC: Epithelial ovarian carcinoma
PFS: Progression-free survival
POC: Primary ovarian cancer
PRISMA: Preferred Reporting Items for Systematic Reviews and Meta-Analysis
QUIPS: Quality in Prognosis Studies
RD: Residual disease
RMI: Risk of Malignancy Index
RPV: Radiomic prognostic vector
RQS: Radiomic Quality Score
RS: Radiomic signature
SNPs: Single nucleotide polymorphisms
SOC: Secondary ovarian cancer
SVM: Support vector machine
T2W: T2 weighted
